# Hormones and Neuropeptide Receptor Heteromers in the Ventral Tegmental Area. Targets for the Treatment of Loss of Control of Food Intake and Substance Use Disorders

**DOI:** 10.1007/s40501-017-0109-x

**Published:** 2017-04-22

**Authors:** Sergi Ferré

**Affiliations:** Integrative Neurobiology Section, National Institute on Drug Abuse, Intramural Research Program, National Institutes of Health, Triad Technology Building, 333 Cassell Drive, Baltimore, MD 21224 USA

**Keywords:** Neuropeptides, Hormones, Ventral tegmental area, Receptor heteromers, Obesity, Substance use disorders

## Abstract

Hormones and neuropeptides represent biological correlates of internal homeostatic signals detected and integrated in the hypothalamus, which establishes a robust functional connection with the ventral tegmental area (VTA). The hypothalamus-VTA connection determines the ability of these signals to influence central dopaminergic neurotransmission and, therefore, their ability to increase responsiveness to their reward-associated stimuli and to establish appropriate associative learning. The hypothalamus also provides the main source of the multiple neuropeptides that are released in the VTA. With volume transmission of neuropeptides and hormones, extrasynaptic receptors within the VTA provide a fine-tune mechanism, which depends on the ability of molecularly different G protein-coupled receptors (GPCRs) to form heteromers. GPCR heteromer is defined as a macromolecular complex composed of at least two different receptor units (protomers) with biochemical properties that are demonstrably different from those of its individual components. GPCR heteromers can provide unique allosteric properties to specific ligands, which provides new avenues for drug development. We have identified specific GPCR heteromers in the VTA that integrate orexin and CRF neurotransmission and opioid and galanin neurotransmission, which play a very significant role in the modulation of dopaminergic neuronal activity and which can constitute targets for the treatment of loss of control of food intake and substance use disorders.

## Introduction: hormones and neuropeptides as interoceptive discriminative stimuli

“Drives” and “motivational states” can be operationally approached by specific *interoceptive discriminative stimuli*, correlative internal signals conveyed by identifiable biological signaling molecules, largely hormones and neuropeptides. For instance, “hunger” and “satiety” can be operationally approached by specific correlative internal orexigenic and anorectic signaling molecules, which respectively facilitate and inhibit food-oriented behavior, eliciting approach to food-related stimuli and consummatory eating behavior or withdrawal from those stimuli. Alterations in the capacity of these biological molecules to signal through their specific receptors lead to pathological implications, such as obesity and anorexia.

*Definitions of hormone and neuropeptide*

*A hormone is defined as a signaling molecule produced by an endocrine cell that is transported by the circulatory system to target distant organs. A neuropeptide is defined as a small proteinaceous substance produced by neurons, released in a regulated fashion and acting on neural substrates, e.g., neurons, glial cells, or non-neuronal target cells, e.g., a gland or muscle* [1].

The hypothalamus represents a key center of integration of homeostatic, threat, and reproductive signals. First, it acts like an *internal sensory organ* that detects internal signals conveyed by hormones and neuropeptides. Second, the hypothalamus “valuates” among the constantly detected internal signals, playing a decision-maker role on the elicitation of the highest priority homeostatic- or reproductive-oriented response.

Several hormones and neuropeptides, such as ghrelin, leptin, insulin, melanocyte-stimulating hormones (α-MSH, β-MSH and γ-MSH), neuropeptide Y, agouti-gene-related protein (AgRP), neurotensin, melanin-concentrating hormone (MCH), orexin, and galanin, provide orexigenic or anorectic signals that are integrated in the hypothalamus to modulate food-oriented behavior. This multiplicity of signals allows an exhaustive control of the internal metabolic environment and more specifically of energy homeostasis [2, 3•, 4•].

**Fig. 1:**
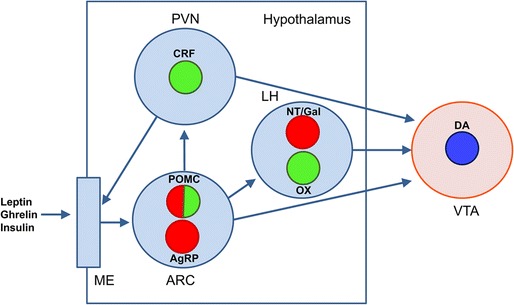
Scheme of intrahypothalamic and hypothalamic-VTA connections. *Green*, *red* and *blue circles* represent glutamate, GABA and dopamine neurons, respectively. *AgRP*, *DA*, *CRF*, *NT*/*Gal*, *POMC*, and *OX* represent neurons expressing aguti-related protein, dopamine, corticotropin-releasing hormone, neurotensin or galanin, pro-opiomelanocortin and orexin, respectively. *ARC* arcuate nucleus, *LH* lateral hypothalamus, *ME* median eminence, *PVN* paraventricular nucleus, *VTA* ventral tegmental area.

Some of the biological signals, particularly ghrelin, leptin, and insulin, are released to the blood circulation and reach the hypothalamic arcuate nucleus (ARC) by way of its proximity to the median eminence [5–7] (Fig. 1). Median eminence is a circumventricular organ with fenestrated capillaries and modified glial cells called tanycytes that play an important part in the neuroendocrine system by providing the connection of hypothalamic neurons to the pituitary, through the pituitary portal circulation [8, 9]. Nerve terminals from the paraventricular nucleus of the hypothalamus (PV) release corticotropin-releasing factor (CRF) within the median eminence, as part of the initial stress response of the hypothalamo-pituitary-adrenocortical axis. CRF then travels to the anterior and intermediate pituitary lobes and promotes the synthesis and systemic release of pro-opiomelanocortin (POMC)-derived peptides, which include the melanocortins adrenocorticotropic hormone (ACTH) and α-, β-, and γ-MSH, as well as the endogenous opioid β-endorphin [3•, 10]. The median eminence acts therefore as a two-way gate for hormones of central and peripheral origin.

Ghrelin is an orexigenic hormone mostly produced by the stomach oxyntic cells, which provide plasma levels that fluctuate diurnally with a peak in the day and through at night [11, 12]. Insulin and glucose independently increases and decreases, respectively, the secretion and circulating concentration of ghrelin [13]. Notably, oxyntic cells qualify as food-entrained oscillators, and ghrelin plasma levels increase during anticipated mealtimes and decrease after meals [11].

Leptin is produced by adipocytes in proportion to triglyceride stores, serving as a signal of repletion of long-term energy stores [14]. The central signals provided by peripheral ghrelin and leptin largely contribute to energy homeostasis, which is achieved when the amount of energy consumed (from food intake) equals energy expended (from basal metabolic rate and physical activity). These signals are initially processed in the hypothalamus, within the ARC, by the two phenotypically different POMC- and AgRP-expressing neurons, which project to the PVN and lateral hypothalamus (LH) (Fig. 1). POMC-derived peptides are therefore not only pituitary-derived hormones, but also neuropeptides synthesized within the hypothalamus. Activation of the POMC-expressing neuron inhibits food intake and food-oriented behaviors, while activation of the AgRP-expressing neuron promotes food intake [4, 15–18].

The orexigenic AgRP-expressing neuron contains high levels of ghrelin receptors. Therefore, ghrelin directly activates AgRP-expressing neurons, which by inhibitory collaterals inhibit the activity of POMC-expressing neurons. On the other hand, both AgRP- and POMC-expressing neurons contain leptin receptors, which stimulate and inhibit the activity of POMC- and AgRP-expressing neurons, respectively [19, 20]. Another level of integration of orexigenic and anorectic signals processed by AgRP- and POMC-expressing neurons takes place at the very melanocortin receptor level. Thus, AgRP is an endogenous orthosteric antagonist of the targets of hypothalamic melanocortins, MC3 and MC4 receptors [3•, 10].

Pathological alterations in the capacity of ghrelin, leptin, and melanocortins to signal through their specific receptors lead to their incapacity to act as appropriate internal homeostatic signals, leading to pathological loss of control of food intake, both in the experimental animal and in human beings. Leptin deficiency and mutations of leptin receptors and MC4 receptors are common monogenic causes of obesity [3•]. Prader-Willi syndrome (PWS), a condition associated with high ghrelin serum levels, is the most common cause of syndromic obesity [21]. PWS patients are incapable of making appropriate food-related decisions and their hyperphagia can be life-threatening. Thus, the voracious feeding habits seen in PWS include intense food foraging, stealing of food and even consumption of inedible foods, which may lead to chocking and gastric rupture [22, 23]. The loss of control of food-related behaviors observed in hyperghrelinemic PWS is a strong and persistent food-oriented psychomotor activation equivalent to that induced by food deprivation, but without satiety. It is therefore equivalent to the psychostimulant effects of addictive drugs, which depend largely on activation of the central dopaminergic system [24]. *The question is then how ghrelin and other food-related internal signals can influence the central dopaminergic system.*

## The hypothalamus-VTA connection

Ascending dopamine systems originate in the substantia nigra pars compacta (SNc) and the ventral tegmental area (VTA), and their differential functional roles depend on their innervation of functionally different striatal compartments as well as their differential source of inputs. The hypothalamus projects mostly to the VTA [25] and in fact constitutes one of its three main sources of inputs, together with the ventral striatopallidal complex and the dorsal raphe [25, 26]. LH is the main contributor of hypothalamic inputs to the VTA, followed by the PV and with a lesser contribution from other nuclei including ARC [25]. Pioneering studies on intracranial electrical stimulation in rodents showed that electrical stimulation of the LH produces voracious feeding behavior [27] as well as reinforcement of lever-pressing behavior to gain additional stimulation, intracranial self-stimulation [28].

It has been classically known that electrical stimulation of the LH facilitates a variety of species-typical, biologically primitive behavior patterns, including eating, drinking and gnawing, in sated animals [28, 30]. Such stimulation does not elicit specific motor responses, but produces psychomotor activation, which implies an increased responsiveness to a variety of environmental stimuli, which implies a facilitation of different behavioral responses, such as feeding, drinking, gnawing of wood, or a predatory attack [29–32]. These differences are not a result of differences in the stimulation region [33] and the dominant response of a given animal changes as a function of the discriminative and rewarding stimuli present [34]. An important amount of data now indicates that most effective intracranial electrical and optogenetic stimulation-induced feeding and reward (intracranial self-stimulation) largely result from the direct activation of VTA dopaminergic cells as well as the LH and the LH-VTA connection, the descending medial forebrain fibers of passage that directly or indirectly activate VTA dopaminergic cells; for review, see [35••].

*It is then through the LH-VTA connection that hypothalamic activity promotes an increased dopaminergic neurotransmission and, consequently, of the dopamine functions: Dopamine release increases responsiveness to rewarding and reward-associated, discriminative stimuli, with orienting and approaching responses to those stimuli* [36]*; concomitantly, dopamine is directly involved in reinforcement, in the learning* (**“** *stamping-in* **”**) *of stimulus-reward and reward-response associations that follows the receipt of reward* [36]*. The reinforcement of stimulus-reward associations establishes new signals that guide and orient to rewards (discriminative stimuli) or which become rewards themselves (conditioned rewarding stimulus). The stamping-in of reward-response associations promotes the learning of the optimal sequential response, the action skill that leads to the reward* [36]*.*

The LH-VTA connection includes glutamatergic and GABAergic projecting neurons that potentially activate and disinhibit (by inhibiting GABAergic interneurons) dopaminergic neuronal activity, respectively [37–41] (Fig. 1). Although it was initially believed that the excitatory input was fundamental to the LH-mediation of dopaminergic cell activation in the VTA [37, 38], recent optogenetic studies have demonstrated a predominant role of the GABAergic projecting neurons in feeding-oriented behavior and reward [39–41]. However, the picture is more complex than just the glutamate-GABA dichotomy, with the existence of different subpopulations of cells expressing different neuropeptides [35••, 42]. For instance, neurotensin and galanin are co-expressed in a population of GABAergic neurons that also express leptin receptors [43, 44] and these neurons exert a local inhibitory control of glutamatergic neurons that co-express orexin [43, 44]. The two orexin/hypocretin peptides, orexin-A and orexin-B, are only produced in the brain by neurons of the lateral hypothalamic nucleus (HL) and to a lesser extent by neurons of the adjacent dorsomedial hypothalamic nucleus [45]. These cells give origin to the ascending orexin arousal system innervating most brain areas, including the VTA [45]. Still another population expresses MCH, which seem to be predominantly GABAergic [46], but these neurons do not connect directly with the VTA [42].

In addition to the LH, VTA receives inputs from other hypothalamic nuclei, such as PV and ARC [25] (Fig. 1). PV provides a main source of CRF to the VTA [47], and ARC provides the source of POMC-derived peptides, melanocortins and β-endorphin [48, 49]. The factors that determine the differential synthesis of POMC-derived peptides are tissue-specific, and the precursor is cleaved in a differential manner. Thus, the processing of POMC in the anterior pituitary is less extensive than in the hypothalamus, where ACTH is all cleaved to produce α-MSH and β-lipotropin is all cleaved to yield β-endorphin [50]. Surprisingly, although the cleavage of POMC to produce α-MSH and β-endorphin depends on the activity of the same enzyme (protein convertase PC2; 50), a recent study showed a cannabinoid CB_1_ receptor-dependent differential synthesis of both neuropeptides by the hypothalamic POMC-expressing neurons [51]. Under normal conditions, activation of POMC-expressing neurons promotes melanocortin synthesis and release, inhibiting food-oriented behavior (see above). However, activation of CB_1_ receptors on POMC-expressing neurons promotes β-endorphin synthesis and the opposite behavioral effect, facilitation of feeding [51]. Two additional opioids, the tetra-peptides endomorphin-1 and endomorphin-2, which are the most potent and selective endogenous agonists for the μ-opioid receptor [52], are also synthesized in hypothalamic neurons which project to the VTA and are localized in an area close to the periventricular nucleus and the ARC [53]. Finally, although still a matter of debate [7], there are data suggesting that ghrelin qualifies as a neuropeptide synthesized in the ARC [54], which could provide an additional ligand source for ghrelin receptors localized in the VTA (see below).

## Hormone and neuropeptide transmission within the VTA

*VTA hormone and neuropeptide GPCRs*

NTS_1_ and NTS_2_ neurotensin receptors [55], Gal_1_ and Gal_2_ receptors [56], OX_1_ and OX_2_ orexin receptors [57], CRF_1_ receptors and lower expression of the CRF_2_ subtype; [58]; melanocortin MC_3_ receptors and lower expression of the MC_4_ subtype; [49]; μ-and κ-opioid receptors [59] and ghrelin GHS_1a_ receptor [60].

Neuropeptide neurotransmission plays then a key role in the hypothalamus-VTA connection. The VTA shows a high density of receptors for neuropeptides localized in the soma and/or dendrites of the dopaminergic cells or in the terminals of their excitatory or inhibitory afferents. Neuropeptide receptors are mostly G protein-coupled receptors (GPCRs), belonging to class A or rhodopsin family (receptors for neurotensin, galanin, orexins, melanocortins, endogenous opioids, and ghrelin) and class B or secretin family (receptors for CRF). Neuropeptides are up to 50 amino acid-long polypeptide gene products, synthesized as ribosomal pre-hormones that are cleaved and often post-translationally modified [61••].

Neuropeptides are therefore produced in the soma and to lesser extent dendrites and packaged in large dense core vesicles that are transported through axons and dendrites. Cleavage and additional enzymatic modifications such as a common C-terminal α-amidation take place in the large dense core vesicles, yielding the final bioactive peptide [61••]. Neuropeptides are released extrasynaptically and preferentially with high-frequency neuronal firing. Classical neurotransmitters, on the other hand, are usually stored in clear synaptic vesicles and released at the synapse upon low-frequency activity. An additional fundamental difference with classical neurotransmitters is that neuropeptide clearance is slower and does not depend on efficient reuptake and intracellular metabolization mechanisms, but on extracellular breaking down by extracellular peptidases. Neuropeptide neurotransmission is therefore designed to reach larger distances than classical neurotransmitters, to exert a modulatory role by activating extrasynaptic receptors localized in different neuronal or non-neuronal elements within a brain area. In general, neuropeptides demonstrate higher affinity and selectivity for their respective receptors than classical neurotransmitters, which corresponds to their ability to act as extrasynaptic modulatory signals. Synaptic transmission, on the other hand, depends on the release of high concentrations of the classical neurotransmitter [61••, 62].

Neuropeptide transmission within the VTA provides a local hormonal-like effect of signaling molecules that are released in a volume-transmission mode [62] from hypothalamic-VTA nerve terminals and invade the extracellular space that surrounds the soma and dendrites of mesencephalic dopaminergic cells. The same neuropeptide-mediated modulatory signal is therefore broadcasted by the bulk of dopaminergic cells to their widespread terminal fields. However, the VTA also contains a relatively high density of hormone receptors, for ghrelin (also GPCRs) and for leptin and insulin (both belonging to the large tyrosine kinase receptor family) [63]. The same as for leptin and ghrelin, insulin can enter the brain tissue by passive diffusion through the median eminence and activate receptors that are localized in the ARC [64], but the three hormones can cross the blood-brain barrier and reach other hypothalamic areas and the VTA by means of specific saturable transport systems [5, 65–67].

Numerous experiments have addressed the study of the effect of each neuropeptide and hormone in isolation on the activity of their specific receptors on VTA dopaminergic cell function. In this way, for instance, it has been established that acting on receptors localized in the dopaminergic cells, ghrelin, neurotensin, or melanocortin increase, while leptin or insulin inhibit their activity [44, 49, 60, 68, 69]. Orexin and opioids also increase dopaminergic cell activity, but most probably by acting on receptors localized in glutamatergic and GABAergic nerve terminals, respectively, increasing excitatory and decreasing inhibitory neurotransmission [70–75]. Less clear has been the role of CRF, which seems to activate dopaminergic cells under specific conditions, such as previous psychostimulant exposure [76], and even more mysterious has been the role of galanin in the VTA [77]. This approach, the investigation of the role of a single neuropeptide or hormone, represents an artificial simplification since the presence of multiple modulators diffusely released in the extracellular space of the VTA is probably the norm, which calls for the analysis of neurotransmitter-neurotransmitter and receptor-receptor interactions. *With volume transmission of neuropeptides and hormones*, *extrasynaptic receptors within the VTA provide an additional molecular fine-tune mechanism*, *which depends on the ability of GPCRs to form oligomeric complexes*, *GPCR heteromers.*

## Hormone and neuropeptide receptor heteromers in the VTA

**Fig. 2:**
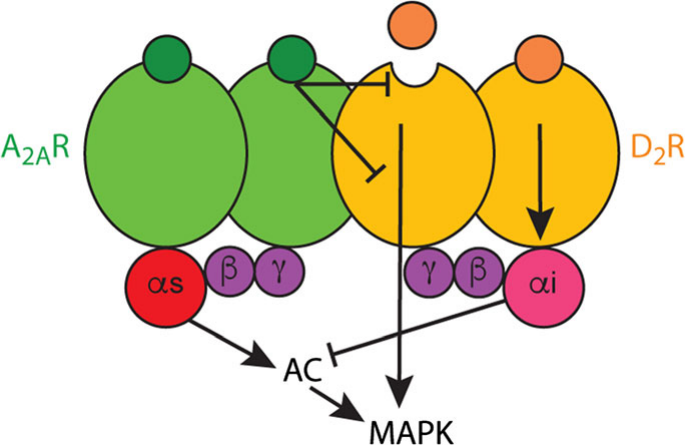
The adenosine A_2A_-dopamine D_2_ receptor heteromer. The heteromer has a heterotetrameric structure, constituted by homodimers of the Gs-coupled A_2A_ receptor (*A*_*2A*_ *R*) and the Gi-coupled D_2_ receptor (*D*_*2*_ *R*). The canonical Gs-Gi antagonistic interaction at the adenylyl cyclase (AC) level and functionally selective allosteric modulation occur in the frame of the A_2A_R-D_2_R heteromer (see text). This heteromer constitutes a predominant population of striatal A_2A_R and D_2_R.

Since their discovery, receptors have mostly been considered as single functional units. However, in recent years, a fast-growing list of GPCR-forming receptor oligomers has emerged [78–80••]. *Receptor heteromer is defined as a macromolecular complex composed of at least two (functional) receptor units (protomers) with biochemical properties that are demonstrably different from those of its individual components* [78]. A first important concept that arises from the new field of GPCR oligomerization is that the pentameric structure constituted by one GPCR homodimer and one heterotrimeric G protein provides a main functional unit, and oligomeric entities can be viewed as multiples of dimers [79••]. This seems to apply particularly to heteromers that include GPCR homodimers with preferential coupling to Gs/olf (Gs for short) proteins and another molecularly different homodimer with preferential coupling to Gi/o (Gi for short) proteins. Such a “GPCR heterotetramer” would sustain a functional pre-coupled macromolecular complex that includes two molecularly different GPCRs, their cognate G proteins, and adenylyl cyclase and would provide the frame for a canonical interaction at the adenylyl cyclase level, the ability of a Gi-coupled GPCR to counteract adenylyl cyclase activation induced by a Gs-coupled GPCR [81]. Recent studies using biophysical techniques and computerized modeling have provided experimental evidence for the existence of several GPCR heterotetramers that fulfill this scheme, such as the dopamine D_1_–D_3_ [82] and the adenosine A_2A_-dopamine D_2_ receptor heterotetramer [83] (Fig. 2). Using disrupting synthetic peptides with the amino acid sequence of different transmembrane domains of the receptors, we can now determine not only the interfaces involved in hetero- and homomerization in the heterotetramer [82, 83] but also the interfaces involved in the complex formation with adenylyl cyclase. One unexpected and very significant output of these studies is that the canonical Gs-Gi interaction at the adenylyl cyclase level is a specific property of a heterotetramer (in preparation).

GPCR heteromers can convey *allosteric modulations between orthosteric ligands* (ligands that bind to the same site as the endogenous neurotransmitter), altering their affinity or intrinsic efficacy. A ligand binding to one GPCR unit in the complex can lead to changes in the properties of a ligand binding to a different GPCR unit [79••]. For instance, in the well-established A_2A_-D_2_ receptor heteromer, an orthosteric A_2A_ receptor agonist decreases the affinity and intrinsic efficacy of dopamine or another orthosteric agonist for the D_2_ receptor [83, 84]. Interestingly, the negative modulation of the intrinsic activity is functionally selective for the G protein-independent D_2_ receptor-mediated MAPK activation [84] (Fig. 2). Although this functional selectivity depends on the existence of neuronal calcium-binding proteins that selectively bind to the A_2A_-D_2_ receptor heteromer. In their absence, knocking down the expression of these calcium-binding proteins, an A_2A_ receptor agonist is also able to counteract the canonical interaction, in this case, the ability of a D_2_ receptor agonist to counteract adenylyl cyclase activation induced by an A_2A_ receptor agonist [84]. A common property of receptor heteromers is that, not only agonists but also antagonists, can act as allosteric modulators within a GPCR heteromer, a phenomenon named cross-antagonism [79••]. Commonly, any orthosteric ligand of one of the protomers in the heteromer can lead to changes in the affinity or intrinsic efficacy of an orthosteric agonist of the molecularly different protomer. This is also the case for the A_2A_-D_2_ receptor heteromer [83].

Additional considerations about the functional and pharmacological properties, which make GPCR attractive targets for drug development, are ligand-independent allosteric modulations and probe dependence. One of the GPCR protomers can convey a ligand-independent allosteric modulation of ligands binding to the other molecularly different GPCR protomer and this can be ligand specific (probe dependence). Again, the A_2A_-D_2_ receptor heteromer provided the proof of concept, since a specific A_2A_ receptor antagonist (SCH-442416) was found to significantly decrease its affinity for the A_2A_ receptor when it heteromerizes with the D_2_ receptor [85].

We have demonstrated the existence of specific GPCR heteromers in the VTA that integrate orexin and CRF neurotransmission [86••] and opioid and galanin neurotransmission [87••], which play a very significant role in the modulation of dopaminergic neuronal activity. Furthermore, we are obtaining experimental evidence for an additional functionally significant GPCR heteromer that controls VTA dopaminergic cell function, a ghrelin-dopamine D_1/5_ receptor heteromer (in preparation). Our methodology includes, first, in vitro techniques in mammalian cells that are transfected with receptors fused to biosensors that can only interact when in close proximity. This allows finding synthetic peptides with amino acid sequences corresponding to the interfaces of the putative receptor heteromers. Specific disrupting peptides are then used as a tool to identify the biochemical properties of the GPCR heteromer (which are specifically disrupted with the peptides), such as an allosteric interaction between specific ligands. Next, the specific disrupting peptides are used with in situ and in vivo approaches to demonstrate the presence of the same GPCR heteromer within the VTA and its functional and pharmacological significance. The approaches include signaling in VTA slices and a modified infusion-microdialysis technique that allows a slow-rate infusion of combinations of neuropeptides and synthetic disruptive peptides and simultaneous measurement of VTA somatodendritic dopamine release [86••]. Somatodendritic dopamine release by mesencephalic dopaminergic cells resembles that of the terminal regions, possessing a similar uptake mechanism and a finite releasable storage pool [88]. Furthermore, previous studies have conclusively shown that local dopamine release in the VTA is a correlate of dopaminergic cell firing [89]. This in vivo approach is therefore particularly suited to explore the role of local neuropeptide interactions within the VTA on dopaminergic cell activity and dependence on GPCR heteromerization.

**Fig. 3:**
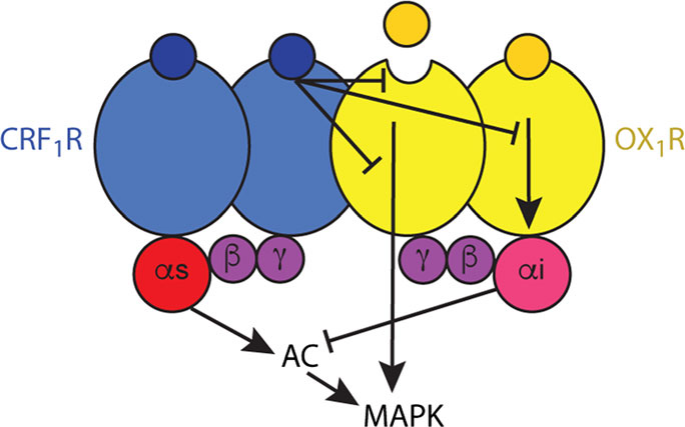
The corticotropin-releasing factor CRF_1_-orexin OX_1_ receptor heteromer. The heteromer can have a heterotetrameric structure, such as the A_2A_-dopamine D_2_ receptor heteromer, constituted by homodimers of a Gs-coupled CRF_1_ receptor (CRF_1_R) and a Gi-coupled OX_1_ receptor (OX_1_R). There is no evidence for functional selectivity of the allosteric interactions within the heteromer. CRF counteracts all signaling events induced by orexin-A, including its potential ability to inhibit CRF-induced AC activation (canonical Gs-Gi interaction; see text).

Specifically, OX_1_ and not OX_2_ receptors form heteromers with CRF_1_ receptors [86••] (Fig. 3). In signaling experiments in transfected cells and in the VTA, CRF_1_-OX_1_ heteromer mediated a strong negative crosstalk between orexin-A and CRF and a cross-antagonism, with the ability of OX_1_ and CRF_1_ receptor antagonists to counteract the effect of CRF_1_ and OX_1_ receptor agonists, respectively. In the CRF_1_-OX_1_ heteromer, CRF_1_ couples to Gs and OX_1_ to Gi, promoting activation and inhibition of adenylyl cyclase, respectively [86••], and therefore probably constituting an additional example of a GPCR heterotetramer [81]. Different to the A_2A_-D_2_ receptor heteromer, the negative allosteric modulation of CRF on the intrinsic efficacy of orexin-A did not show functional selectivity. CRF counteracted both signaling events induced by orexin-A, the G protein-dependent ability of orexin to counteract CRF1 receptor-mediated adenylyl cyclase activation, and the G protein-independent ability to activate MAPK activation [86••] (Fig. 3). Orexin-A produced a significant increase in somatodendritic dopamine release in the VTA and CRF was not effective on its own, but significantly counteracted the effect of orexin-A. Cross-antagonism could also be demonstrated, and a CRF receptor antagonist also counteracted the effect of orexin-A [86••]. That these pharmacological interactions were dependent on CRF_1_-OX_1_ heteromerization was demonstrated by the ability of heteromer-specific peptides to disrupt the negative crosstalk in vivo [86••]. CRF-orexin-A interactions mediated by the CRF_1_-OX_1_ heteromers could be an extension of the integrative role of internal signals by the hypothalamus. The CRF_1_-OX_1_ heteromers integrate volume transmission signals driven by neuropeptides from the PV and LH, probably under conditions where the metabolic demands that facilitate food-oriented behavior need to be inhibited in favor of more evolutionary-significant behaviors, such as those linked to threat signals.

An additional pharmacological finding of the CRF_1_-OX_1_ heteromer was that it complexes with sigma σ_1_ receptors and that σ_1_ receptor ligands, including cocaine, counteract the allosteric interactions within the heteromer, both in transfected cells and in the VTA. With the infusion-microdialysis technique, it could also be shown that under normal conditions, the CRF_1_-OX_1_ heteromer mediates a tonic inhibitory influence of orexin-A on CRF-induced signaling that can be released by cocaine exposure [86••]. The counteraction of the negative crosstalk between orexin-A and CRF in the VTA by σ_1_ receptor ligands provides a mechanism by which CRF can only induce glutamate-dependent somatodendritic dopamine release in animals previously exposed to cocaine [76]. Counteraction of the allosteric interactions between the endogenous neuropeptides in the CRF_1_-OX_1_ receptor heteromer can also explain the previously reported apparent CRF-independent ability of orexin-A to release dopamine in the VTA and to induce cocaine seeking [90]. The localization of CRF_1_-OX_1_ heteromers in the VTA still remains to be determined, although the previously reported glutamate dependence of the somatodendritic dopamine release in the VTA induced by orexin-A or stress strongly suggests their localization in the glutamatergic terminals of PVN-VTA neurons [76, 90]. CRF_1_-OX_1_ receptor heteromer can therefore constitute a target for the treatment of cocaine and other psychostimulant use disorders.

Galanin receptors have been said to provide candidates for the treatment of opioid use disorders [91]. This is based, first, on experimental evidence for the existence of antagonistic interactions between the galanin and opioid systems. Second, on genetic studies that find associations of galanin gene polymorphisms with susceptibility to opioid use disorder [92, 93]. The most significant association was observed for the single nucleotide polymorphism rs948854, localized in the promoter region [93]. The experimental results indicate that galanin receptor activation counteracts the psychomotor-activating and reinforcing effects of morphine [91, 94]. The behavioral effects of morphine were enhanced in galanin knock-out mice and were also counteracted by the systemic administration of a non-peptidergic, non-selective galanin receptor agonist [91]. Significantly, galanin knock-out mice showed a selective increase in morphine-induced MAPK activation in the VTA, which was also counteracted by the galanin receptor agonist [91].

**Fig. 4:**
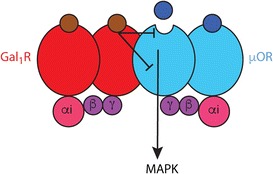
The μ-opioid-galanin Gal_1_ receptor heteromer. The heteromer can have a heterotetrameric structure, constituted by homodimers of two Gi-coupled receptors, the μ-opioid (μOR) and the Gal_1_ receptor (Gal_1_R). Within the heteromer, galanin exerts a strong negative allosteric control of μOR-mediated MAPK signaling (its G protein dependence or independence still needs to be determined). This heteromer constitutes a predominant population of μOR localized in the VTA.

It is well established that μ-opioid receptors localized in the striatum and in the ventral midbrain are involved in the reinforcing effects of opioids [94–97]. In the ventral midbrain, endogenous opioids exert a strong inhibitory control of the function of dopaminergic cells in the VTA, which depends on μ-opioid-mediated inhibition of a tonic GABAergic neurotransmission [70], largely mediated by afferents from the striatal patch compartment and from the rostromedial tegmental nucleus or tail of the VTA [72, 73, 75]. We recently demonstrated the existence of heteromers of μ-opioid and, specifically, Gal_1_ receptors in the VTA that can underlie the pharmacological interactions of opioids and galanin systems [87••] (Fig. 4). These μ-opioid-Gal_1_ receptor heteromers are therefore most probably localized in inhibitory inputs to the VTA and integrate volume transmission signals driven by the hypothalamic inputs that release β-endorphin and endomorphin-1 (ARC and periARC and periventricular areas) and galanin (LH). A hormonal pituitary origin of β-endorphin seems to be unlikely [98].

We first detected μ-opioid-Gal_1_ receptor heteromerization in mammalian transfected cells and obtained a specific peptide that disrupted the heteromerization and a negative crosstalk, by which galanin counteracted endomorphin-1-mediated MAPK activation [87••] (Fig. 4). The negative crosstalk constituted therefore a biochemical property of μ-opioid-Gal_1_ heteromer, which could also be identified in situ in VTA slices and in vivo with microdialysis experiments. Thus, galanin completely counteracted somatodendritic dopamine release induced by the local infusion of endomorphin-1. Both in situ and in vivo galanin-opioid interactions were also selectively counteracted by application of the disruptive peptide, demonstrating their dependence on μ-opioid-Gal_1_ heteromerization [87••]. These results indicate that dopaminergic cell function in the VTA is modulated by a predominant population of μ-opioid receptors forming heteromers with Gal_1_ receptors. Therefore, μ-opioid-Gal_1_ receptor heteromers constitute an obvious target for the treatment of opioid use disorders.

**Fig. 5:**
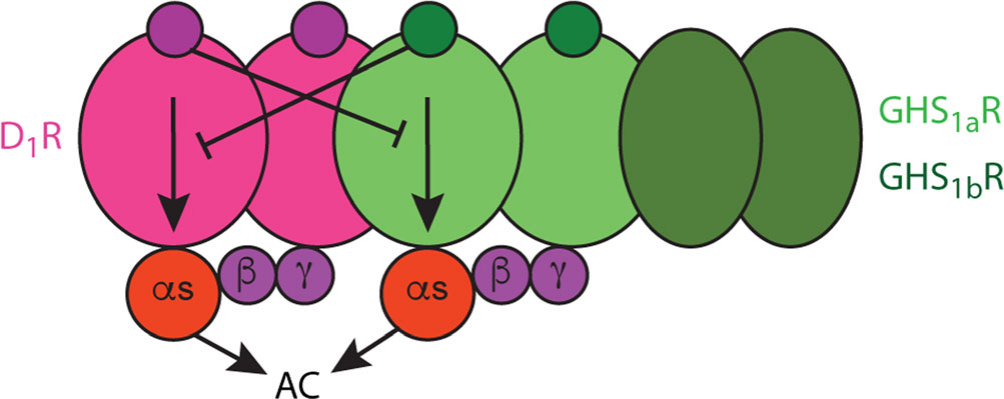
The ghrelin GHS_1a_-GHS_1b_-dopamine D_1_ receptor heteromer. Oligomerization with the truncated ghrelin GHS_1b_ receptor (GHS_1b_R) allows GHS_1a_R to heteromerize with dopamine D_1_-like receptors (D_1_R or D_5_R) and couple to Gs protein. Activation of AC becomes therefore a main signaling pathway of the heteromer. Within the heteromer, dopamine and ghrelin receptor ligands establish negative allosteric interactions. This heteromer has been demonstrated in striatal cells in culture, and indirect evidence indicates that it is also functionally present in the VTA.

There is also indirect evidence for the existence of ghrelin receptor heteromers and neurotensin receptor heteromers localized in the dopaminergic cells, although they do not fulfill yet the criteria for their identification within the VTA [78–19, 80••]. Ghrelin receptors are known as growth hormone secretagogue (GHS) receptor or GHS_1a_ receptors. Cells expressing GHS_1a_ also express GHS_1b_ receptors, a truncated variant of GHS_1a_ receptors lacking the transmembrane domains 6 and 7. Ghrelin does not bind and therefore does not signal through GHS_1b_ receptors [99] and the role of this truncated “receptor” on ghrelin-mediated signaling is just beginning to be understood. Evidence has been provided for the ability of GHS_1a_ to homodimerize and to heterodimerize with GHS_1a_ receptors, which allows GHS_1b_ to produce a dominant negative effect on GHS_1a_ receptor signaling [99, 100]. Using mammalian transfected cells and neuronal cells in culture, we found, first, a significant and complex modulatory role of GHS_1b_ in the trafficking and signaling of GHS_1a_ receptors that depends on the relative expression of both proteins [101]. An additional finding in striatal and hippocampal neurons in culture was a predominant Gs/olf protein-dependent signaling of ghrelin, which in striatal neurons depended on D_1_-GHS_1a_-GHS_1b_ receptor heteromerization [101] (Fig. 5). A D_1_ receptor antagonist blocked ghrelin-induced cAMP accumulation in striatal but not hippocampal neurons, indicating the involvement of D_1_ receptors in the striatal GHS_1a_-Gs/olf coupling. Experiments in transfected cells demonstrated that D_1_ receptor co-expression promotes a switch in GHS_1a_-G protein coupling, from Gi/o to Gs/olf, but only upon co-expression of GHS_1b_. In fact, with biophysical techniques (resonance energy transfer experiments), it could be demonstrated that D_1_ receptor interacts with GHS_1a_, but only in the presence of GHS_1b_ [101]. Finally, a negative crosstalk could also be observed upon co-administration of D_1_ and GHS_1a_ receptor agonists [101] (Fig. 5). Therefore, GHS_1b_ not only determines the efficacy of ghrelin-induced GHS_1a_-mediated signaling but also determines the ability of GHS_1b_ to form oligomeric complexes with other receptors promoting profound qualitative changes in ghrelin-induced signaling.

Now, there is evidence for the localization of both GHS_1a_ and D_1_-like receptors (D_1_ or D_5_ receptor subtype) in the VTA dopaminergic cells [102]. It is therefore very likely that these neurons will also express D_1/5_-GHS_1a_-GHS_1b_ receptor heteromers. In fact, using the infusion-microdialysis technique, we have obtained evidence for a strong ghrelin-induced somatodendritic dopamine release that is counteracted by co-perfusion with a D_1_ receptor antagonist (in preparation). In addition, GHS_1a_ receptors could also oligomerize with melanocortin receptors in the VTA, as the predominant subtype expressed by dopaminergic neurons, MC_3_ receptors [49], has been reported to form heteromers with GHS_1a_ in the hypothalamic adenylyl cyclase (AC) [103]. Ghrelin receptor heteromers in the VTA could specifically provide new therapeutic targets for obesity associated with high ghrelin serum levels, such as PWS [21]. Finally, NT_1_-D_2_ receptor heteromers [104, 105] would also be localized in dopaminergic cells and could mediate the activating effects of neurotensin related to disinhibition of D_2_ autoreceptor function [44]. NT_1_-D_2_ receptor heteromers would therefore provide an additional disinhibitory mechanism of dopaminergic cell activity provided by GABA-neurotensin-expressing HL neurons.

## Conclusions

The hypothalamic-VTA connection provides a link between the center of integration of internal homeostatic, threat and reproductive signals, and dopaminergic neurotransmission, which determines the degree of psychomotor activation and reinforcing effects elicited by those stimuli. Apart from classical neurotransmitters, the very heterogeneous hypothalamic-VTA connection releases a series of neuropeptides in a volume-transmission mode which, together with several hormones that seem to be able to cross the blood-brain barrier within the VTA by saturable trasporters, simultaneously influences the activity of dopaminergic cells. We introduce first the concept of targeting receptors for hormones and neuropeptides localized in the VTA as a therapeutic approach for the loss of control of food intake and substance use disorders. Second, we introduce the concept that the specific targeting of those receptors can be achieved by targeting specific VTA GPCR heteromers.

## References

[1] Burbach JP. Neuropeptides from concept to online database www.neuropeptides.nl. Eur J Pharmacol 2010;626:27–48. 10.1016/j.ejphar.2009.10.015 19837055

[2] Parker JA, Bloom SR (2012). Hypothalamic neuropeptides and the regulation of appetite. Neuropharmacology.

[3] Yeo GS, Heisler LK (2012). Unraveling the brain regulation of appetite: lessons from genetics. Nat Neurosci.

[4] Waterson MJ, Horvath TL (2015). Neuronal regulation of energy homeostasis: beyond the hypothalamus and feeding. Cell Metab.

[5] Münzberg H (2008). Differential leptin access into the brain. A hierarchical organization of hypothalamic leptin target sites?. Physiol Behav.

[6] Schaeffer M, Langlet F, Lafont C, Molino F, Hodson DJ, Roux T, Lamarque L (2013). Rapid sensing of circulating ghrelin by hypothalamic appetite-modifying neurons. Proc Natl Acad Sci U S A.

[7] Cabral A, De Francesco PN, Perello M (2015). Brain circuits mediating the orexigenic action of peripheral ghrelin: narrow gates for a vast kingdom. Front Endocrinol.

[8] Kiss A (1997). Morphological aspects of the median eminence. Place of accumulation and secretion of regulatory neurohormones and neuropeptides. Gen Physiol Biophys.

[9] Krisch B, Leonhardt H (1978). The functional and structural border of the neurohemal region of the median eminence. Cell Tissue Res.

[10] Catania A, Gatti S, Colombo G, Lipton JM (2004). Targeting melanocortin receptors as a novel strategy to control inflammation. Pharmacol Rev.

[11] Silver R, Balsam P (2010). Oscillators entrained by food and the emergence of anticipatory timing behaviors. Sleep Biol Rhythms.

[12] Mason BL, Wang Q, Zigman JM (2014). The central nervous system sites mediating the orexigenic actions of ghrelin. Annu Rev Physiol.

[13] van der Lely AJ, Tschöp M, Heiman ML, Ghigo E (2004). Biological, physiological, pathophysiological, and pharmacological aspects of ghrelin. Endocr Rev.

[14] Sutton AK, Myers MG, Olson DP (2016). The role of PVH circuits in leptin action and energy balance. Annu Rev Physiol.

[15] Aponte Y, Atasoy D, Sternson SM (2011). AGRP neurons are sufficient to orchestrate feeding behavior rapidly and without training. Nat Neurosci.

[16] Krashes MJ, Koda S, Ye C, Rogan SC, Adams AC, Cusher DS (2011). Rapid, reversible activation of AgRP neurons drives feeding behavior in mice. J Clin Invest.

[17] Betley JN, Cao ZF, Ritola KD, Sternson SM (2013). Parallel, redundant circuit organization for homeostatic control of feeding behavior. Cell.

[18] Chen Y, Lin YC, Kuo TW, Knight ZA (2015). Sensory detection of food rapidly modulates arcuate feeding circuits. Cell.

[19] Yang Y, Atasoy D, Su HH, Sternson SM (2011). Hunger states switch a flip-flop memory circuit via a synaptic AMPK-dependent positive feedback loop. Cell.

[20] Cowley MA, Smart JL, Rubinstein M, Cerdán MG, Diano S, Horvath TL, Cone RD, Low MJ (2001). Leptin activates anorexigenic POMC neurons through a neural network in the arcuate nucleus. Nature.

[21] Cummings DE, Clement K, Purnell JQ, Vaisse C, Foster KE, Frayo RS (2002). Elevated plasma ghrelin levels in Prader Willi syndrome. Nat Med.

[22] Stevenson DA, Heinemann J, Angulo M, Butler MG, Loker J, Rupe N (2007). Gastric rupture and necrosis in Prader-Willi syndrome. J Pediatr Gastroenterol Nutr.

[23] Stevenson DA, Heinemann J, Angulo M, Butler MG, Loker J, Rupe N (2007). Deaths due to choking in Prader-Willi syndrome. Am J Med Genet.

[24] Wise RA, Bozarth MA (1987). A psychomotor stimulant theory of addiction. Psychol Rev.

[25] Watabe-Uchida M, Zhu L, Ogawa SK, Vamanrao A, Uchida N (2012). Whole-brain mapping of direct inputs to midbrain dopamine neurons. Neuron.

[26] Faget L, Osakada F, Duan J, Ressler R, Johnson AB, Proudfoot JA (2016). Afferent inputs to neurotransmitter-defined cell types in the ventral tegmental area. Cell Rep.

[27] Delgado JM, Anand BK (1953). Increase of food intake induced by electrical stimulation of the lateral hypothalamus. Am J Phys.

[28] Hess WR (1957). The functional organization of the diencephalon.

[29] Roberts WW, Carey RJ (1965). Rewarding effect of performance of gnawing aroused by hypothalamic stimulation in the rat. J Comp Physiol Psychol.

[30] Glickman SE, Schiff BB (1967). A biological theory of reinforcement. Psychol Rev.

[31] Mogenson GJ, Stevenson JA (1967). Drinking induced by electrical stimulation of the lateral hypothalamus. Exp Neurol.

[32] Coons EE, Levak M, Miller NE (1965). Lateral hypothalamus: learning of food-seeking response motivated by electrical stimulation. Science.

[33] Wise RA (1971). Individual differences in effects of hypothalamic stimulation: the role of stimulation locus. Physiol Behav.

[34] Valenstein ES, Cox VC, Kakolewski JW (1968). Modification of motivated behavior elicited by electrical stimulation of the hypothalamus. Science.

[35] Stuber GD, Wise RA (2016). Lateral hypothalamic circuits for feeding and reward. Nat Neurosci.

[36] Wise RA (2004). Dopamine, learning and motivation. Nat Rev Neurosci.

[37] You ZB, Chen YQ, Wise RA (2001). Dopamine and glutamate release in the nucleus accumbens and ventral tegmental area of rat following lateral hypothalamic self-stimulation. Neuroscience.

[38] Kempadoo KA, Tourino C, Cho SL, Magnani F, Leinninger GM, Stuber GD (2013). Hypothalamic neurotensin projections promote reward by enhancing glutamate transmission in the VTA. J Neurosci.

[39] Jennings JH, Ung RL, Resendez SL, Stamatakis AM, Taylor JG, Huang J (2015). Visualizing hypothalamic network dynamics for appetitive and consummatory behaviors. Cell.

[40] Nieh EH, Vander Weele CM, Matthews GA, Presbrey KN, Wichmann R (2016). Inhibitory input from the lateral hypothalamus to the ventral tegmental area disinhibits dopamine neurons and promotes behavioral activation. Neuron.

[41] Barbano MF, Wang HL, Morales M, Wise RA (2016). Feeding and reward are differentially induced by activating gabaergic lateral hypothalamic projections to VTA. J Neurosci.

[42] Brown JA, Woodworth HL, Leinninger GM (2015). To ingest or rest? Specialized roles of lateral hypothalamic area neurons in coordinating energy balance. Front Syst Neurosci.

[43] Leinninger GM, Opland DM, Jo YH, Faouzi M, Christensen L, Cappellucci LA (2011). Leptin action via neurotensin neurons controls orexin, the mesolimbic dopamine system and energy balance. Cell Metab.

[44] Patterson CM, Wong JM, Leinninger GM, Allison MB, Mabrouk OS, Kasper CL (2015). Ventral tegmental area neurotensin signaling links the lateral hypothalamus to locomotor activity and striatal dopamine efflux in male mice. Endocrinology.

[45] de Lecea L, Kilduff TS, Peyron C, Gao X, Foye PE, Danielson PE (1998). The hypocretins: hypothalamus-specific peptides with neuroexcitatory activity. Proc Natl Acad Sci U S A.

[46] Jego S, Glasgow SD, Herrera CG, Ekstrand M, Reed SJ, Boyce R, Friedman J, Burdakov D, Adamantidis AR (2013). Optogenetic identification of a rapid eye movement sleep modulatory circuit in the hypothalamus. Nat Neurosci.

[47] Rodaros D, Caruana DA, Amir S, Stewart J (2007). Corticotropin-releasing factor projections from limbic forebrain and paraventricular nucleus of the hypothalamus to the region of the ventral tegmental area. Neuroscience.

[48] Jarjour S, Bai L, Gianoulakis C (2009). Effect of acute ethanol administration on the release of opioid peptides from the midbrain including the ventral tegmental area. Alcohol Clin Exp Res.

[49] Pandit R, Omrani A, Luijendijk MC, de Vrind VA, Van Rozen AJ, Ophuis RJ (2016). Melanocortin 3 receptor signaling in midbrain dopamine neurons increases the motivation for food reward. Neuropsychopharmacology.

[50] Bicknell AB (2008). The tissue-specific processing of pro-opiomelanocortin. J Neuroendocrinol.

[51] Koch M, Varela L, Kim JG, Kim JD, Hernández-Nuño F, Simonds SE (2015). Hypothalamic POMC neurons promote cannabinoid-induced feeding. Nature.

[52] Zadina JE, Hackler L, Ge LJ, Kastin AJ (1997). A potent and selective endogenous agonist for the mu-opiate receptor. Nature.

[53] Greenwell TN, Zangen A, Martin-Schild S, Wise RA, Zadina JE (2002). Endomorphin-1 and -2 immunoreactive cells in the hypothalamus are labeled by fluoro-gold injections to the ventral tegmental area. J Comp Neurol.

[54] Mondal MS, Date Y, Yamaguchi H, Toshinai K, Tsuruta T, Kangawa K (2005). Identification of ghrelin and its receptor in neurons of the rat arcuate nucleus. Regul Pept.

[55] Vincent JP, Mazella J, Kitabgi P (1999). Neurotensin and neurotensin receptors. Trends Pharmacol Sci.

[56] Lang R, Gundlach AL, Holmes FE, Hobson SA, Wynick D, Hökfelt T (2015). Physiology, signaling, and pharmacology of galanin peptides and receptors: three decades of emerging diversity. Pharmacol Rev.

[57] Marcus JN, Aschkenasi CJ, Lee CE, Chemelli RM, Saper CB, Yanagisawa M (2001). Differential expression of orexin receptors 1 and 2 in the rat brain. J Comp Neurol.

[58] Wise RA, Morales M (2010). A ventral tegmental CRF-glutamate-dopamine interaction in addiction. Brain Res.

[59] Mansour A, Khachaturian H, Lewis ME, Akil H, Watson SJ (1988). Anatomy of CNS opioid receptors. Trends Neurosci.

[60] Abizaid A, Liu ZW, Andrews ZB, Shanabrough M, Borok E (2006). Ghrelin modulates the activity and synaptic input organization of midbrain dopamine neurons while promoting appetite. J Clin Invest.

[61] Hökfelt T, Bartfai T, Bloom F (2003). Neuropeptides: opportunities for drug discovery. Lancet Neurol.

[62] Agnati LF, Guidolin D, Guescini M, Genedani S, Fuxe K (2010). Understanding wiring and volume transmission. Brain Res Rev.

[63] Figlewicz DP, Evans SB, Murphy J, Hoen M, Baskin DG (2003). Expression of receptors for insulin and leptin in the ventral tegmental area/substantia nigra (VTA/SN) ofthe rat. Brain Res.

[64] Niswender KD, Schwartz MW (2003). Insulin and leptin revisited: adiposity signals with overlapping physiological and intracellular signaling capabilities. Front Neuroendocrinol.

[65] Banks WA, Tschöp M, Robinson SM, Heiman ML (2002). Extent and direction of ghrelin transport across the blood-brain barrier is determined by its unique primary structure. J Pharmacol Exp Ther.

[66] Banks WA (2012). Role of the blood-brain barrier in the evolution of feeding and cognition. Ann N Y Acad Sci.

[67] Woods SC, Seeley RJ, Baskin DG, Schwartz MW (2003). Insulin and the blood-brain barrier. Curr Pharm Des.

[68] Stowe ZN, Nemeroff CB (1991). The electrophysiological actions of neurotensin in the central nervous system. Life Sci.

[69] Labouèbe G, Liu S, Dias C, Zou H, Wong JC, Karunakaran S (2013). Insulin induces long-term depression of ventral tegmental area dopamine neurons via endocannabinoids. Nat Neurosci.

[70] Chefer VI, Denoroy L, Zapata A, Shippenberg TS (2009). Mu opioid receptor modulation of somatodendritic dopamine overflow: GABAergic and glutamatergic mechanisms. Eur J Neurosci.

[71] Borgland SL, Ungless MA, Bonci A (2010). Convergent actions of orexin/hypocretin and CRF on dopamine neurons: emerging players in addiction. Brain Res.

[72] Barrot M, Sesack SR, Georges F, Pistis M, Hong S, Jhou TC (2012). Braking dopamine systems: a new GABA master structure for mesolimbic and nigrostriatal functions. J Neurosci.

[73] Cui Y, Ostlund SB, James AS, Park CS, Ge W, Roberts KW (2014). Targeted expression of μ-opioid receptors in a subset of striatal direct-pathway neurons restores opiate reward. Nat Neurosci.

[74] Mahler SV, Smith RJ, Aston-Jones G (2013). Interactions between VTA orexin and glutamate in cue-induced reinstatement of cocaine seeking in rats. Psychopharmacology.

[75] Matsui A, Jarvie BC, Robinson BG, Hentges ST, Williams JT (2014). Separate GABA afferents to dopamine neurons mediate acute action of opioids, development of tolerance, and expression of withdrawal. Neuron.

[76] Wang B, Shaham Y, Zitzman D, Azari S, Wise RA, You ZB (2005). Cocaine experience establishes control of midbrain glutamate and dopamine by corticotropin-releasing factor: a role in stress-induced relapse to drug seeking. J Neurosci.

[77] Picciotto MR (2008). Galanin and addiction. Cell Mol Life Sci.

[78] Ferré S, Baler R, Bouvier M, Caron MG, Devi LA, Durroux T (2009). Building a new conceptual framework for receptor heteromers. Nat Chem Biol.

[79] Ferré S, Casadó V, Devi LA, Filizola M, Jockers R, Lohse MJ (2014). G protein-coupled receptor oligomerization revisited: functional and pharmacological perspectives. Pharmacol Rev.

[80] Gomes I, Ayoub MA, Fujita W, Jaeger WC, Pfleger KD, Devi LA (2016). G protein-coupled receptor heteromers. Annu Rev Pharmacol Toxicol.

[81] Ferré S (2015). The GPCR heterotetramer: challenging classical pharmacology. Trends Pharmacol Sci.

[82] Guitart X, Navarro G, Moreno E, Yano H, Cai NS, Sánchez-Soto M (2014). Functional selectivity of allosteric interactions within G protein-coupled receptor oligomers: the dopamine D1-D3 receptor heterotetramer. Mol Pharmacol.

[83] Bonaventura J, Navarro G, Casadó-Anguera V, Azdad K, Rea W, Moreno E (2015). Allosteric interactions between agonists and antagonists within the adenosine A2A receptor-dopamine D2 receptor heterotetramer. Proc Natl Acad Sci U S A.

[84] Navarro G, Aguinaga D, Moreno E, Hradsky J, Reddy PP, Cortés A (2014). Intracellular calcium levels determine differential modulation of allosteric interactions within G protein-coupled receptor heteromers. Chem Biol.

[85] Orru M, Bakešová J, Brugarolas M, Quiroz C, Beaumont V, Goldberg SR (2011). Striatal pre- and postsynaptic profile of adenosine A(2A) receptor antagonists. PLoS One.

[86] Navarro G, Quiroz C, Moreno-Delgado D, Sierakowiak A, McDowell K, Moreno E (2015). Orexin-corticotropin-releasing factor receptor heteromers in the ventral tegmental area as targets for cocaine. J Neurosci.

[87] Moreno E, Quiroz C, Rea W, Cai NS, Mallol J, Cortés A (2017). Functional μ-opioid-galanin receptor heteromers in the ventral tegmental area. J Neurosci.

[88] Kita JM, Kile BM, Parker LE, Wightman RM (2009). In vivo measurement of somatodendritic release of dopamine in the ventral tegmental area. Synapse.

[89] Legault M, Wise RA (1999). Injections of N-methyl-D-aspartate into the ventral hippocampus increase extracellular dopamine in the ventral tegmental area and nucleus accumbens. Synapse.

[90] Wang B, You ZB, Wise RA (2009). Reinstatement of cocaine seeking by hypocretin (orexin) in the ventral tegmental area: independence from the local corticotropin-releasing factor network. Biol Psychiatry.

[91] Hawes JJ, Brunzell DH, Narasimhaiah R, Langel U, Wynick D, Picciotto MR (2008). Galanin protects against behavioral and neurochemical correlates of opiate reward. Neuropsychopharmacology.

[92] Levran O, Londono D, O’Hara K, Nielsen DA, Peles E, Rotrosen J (2008). Genetic susceptibility to heroin addiction: a candidate gene association study. Genes Brain Behav.

[93] Beer B, Erb R, Pavlic M, Ulmer H, Giacomuzzi S, Riemer Y (2013). Association of polymorphisms in pharmacogenetic candidate genes (OPRD1, GAL, ABCB1, OPRM1) with opioid dependence in European population: a case-control study. PLoS One.

[94] Wise RA (1989). Opiate reward: sites and substrates. Neurosci Biobehav Rev.

[95] McBride WJ, Murphy JM, Ikemoto S (1999). Localization of brain reinforcement mechanisms: intracranial self-administration and intracranial place-conditioning studies. Behav Brain Res.

[96] Zangen A, Ikemoto S, Zadina JE, Wise RA (2002). Rewarding and psychomotor stimulant effects of endomorphin-1: anteroposterior differences within the ventral tegmental area and lack of effect in nucleus accumbens. J Neurosci.

[97] Jhou TC, Xu SP, Lee MR, Gallen CL, Ikemoto S (2012). Mapping of reinforcing and analgesic effects of the mu opioid agonist endomorphin-1 in the ventral midbrain of the rat. Psychopharmacology.

[98] Pardridge WM, Triguero D, Buciak JL (1990). Beta-endorphin chimeric peptides: transport through the blood-brain barrier in vivo and cleavage of disulfide linkage by brain. Endocrinology.

[99] Mary S, Fehrentz JA, Damian M, Gaibelet G, Orcel H, Verdié P (2013). Heterodimerization with its splice variant blocks the ghrelin receptor 1a in a non-signaling conformation: a study with a purified heterodimer assembled into lipid discs. J Biol Chem.

[100] Leung PK, Chow KB, Lau PN, Chu KM, Chan CB, Cheng CH (2007). The truncated ghrelin receptor polypeptide (GHS-R1b) acts as a dominant-negative mutant of the ghrelin receptor. Cell Signal.

[101] Navarro G, Aguinaga D, Angelats E, Medrano M, Moreno E, Mallol J (2016). A significant role of the truncated ghrelin receptor GHS-R1b in ghrelin-induced signaling in neurons. J Biol Chem.

[102] Skibicka KP, Hansson C, Egecioglu E, Dickson SL (2012). Role of ghrelin in food reward: impact of ghrelin on sucrose self-administration and mesolimbic dopamine and acetylcholine receptor gene expression. Addict Biol.

[103] Rediger A, Piechowski CL, Yi CX, Tarnow P, Strotmann R, Grüters A (2011). Mutually opposite signal modulation by hypothalamic heterodimerization of ghrelin and melanocortin-3 receptors. J Biol Chem.

[104] Koschatzky S, Gmeiner P (2012). Selective agonists for dopamine/neurotensin receptor heterodimers. ChemMedChem.

[105] Hübner H, Schellhorn T, Gienger M, Schaab C, Kaindl J, Leeb L (2016). Structure-guided development of heterodimer-selective GPCR ligands. Nat Commun.

